# Complete mitochondrial genome of the freshwater monogonont rotifer *Brachionus calyciflorus* (Rotifera, Brachionidae)

**DOI:** 10.1080/23802359.2019.1676679

**Published:** 2019-10-16

**Authors:** Beom-Soon Choi, Young Hwan Lee, Atsushi Hagiwara, Jae-Seong Lee

**Affiliations:** a Phyzen Genomics Institute, Seongnam, South Korea;; b Department of Biological Science, College of Science, Sungkyunkwan University, Suwon, South Korea;; c Institute of Integrated Science and Technology, Nagasaki University, Nagasaki, Japan;; d Organization for Marine Science and Technology, Nagasaki University, Nagasaki, Japan

**Keywords:** Monogonont rotifer, complete mitochondrial genome, *Brachionus calyciflorus*, the Netherlands strain

## Abstract

The two complete mitochondrial genomes were sequenced from the Netherlands strain of the freshwater monogonont rotifer *Brachionus calyciflorus*. The mitochondrial genome sequences were 27,698 bp and 9,906 bp in size, respectively. The gene order and contents of the two *B. calyciflorus* strains were mostly identical to one another, except for the additional identification and translocation of several tRNAs in mitochondrial DNA I and II. Of 13 protein-coding genes (PCGs), three genes (*ND1*, *ND5*, and *ND3*) had incomplete stop codons. Furthermore, the start codon of *ND2*, *CO2*, and *CO3* and *ND4* genes was ATT, GTG, and ATA, respectively, while the start codon of other PCGs was ATG. The base composition of 13 PCGs of *B. calyciflorus* (the Netherlands strain) mitogenome showed 31.1% for A, 37.6% for T, 16.5% for C, and 14.8% for G, respectively.

In the freshwater rotifer *Brachionus calyciflorus* species complex, four and eight cryptic species were identified from Netherlands (Papakostas et al. 2016; Michaloudi et al. 2018) and China (Xiang et al. 2011), respectively, as *Brachionus plicatilis* species complex were identified from 15 species (Mills et al. 2017). However, to date, only one complete mitochondrial genome of *B. calyciflorus* (China) has been published (Nie et al. 2016), while several complete mitochondrial genome of other brackish *Brachionus* rotifers have been published; *B. plicatilis* (Suga et al. 2008), *B. koreanus* (Hwang et al. 2013; Hwang et al. 2014), and *B. rotundiformis* (Kim et al. 2017). Thus, the identification of *B. calyciflorus* species complex can be important to better understand the phylogenetic relationship of the freshwater rotifer *B. calyciflorus* species complex clade. Also, recently *B. calyciflorus* is considered as a model for environmental toxicology in response to environmental stressors (Zhang et al. 2015; Nys et al. 2016; Paraskevopoulou et al. 2018; Kim et al. 2018; Lee et al. 2019). The analysis of *B. calyciflorus* mitochondrial genome is important to identify and compare the species-specificity of the field-sampled and laboratory stocks. In this study, we identified two complete mitochondrial genomes of the monogonont rotifer *B. calyciflorus* (the Netherlands).

The resting eggs of *B. calyciflorus* were collected from sediments of freshwater ponds at in November 2015 (kindly provided by Dr. Steven A.J. Declerck, Netherlands Institute of Ecology, the Netherlands) in Zwartenhoek, the Netherlands (52°02′63″N and 4°18′35.5″E) and maintained at the Laboratory of Professor Atsushi Hagiwara, Nagasaki University in Japan. The type was deposited in the Ichthyological collection of the Faculty of Fisheries, Nagasaki University (FFNU) under the accession no. FFNU-Rot-0005. We sequenced 500 bp paired end library of *B. calyciflorus* from whole body genomic DNA using the Illumina HiSeq 2500 platform (GenomeAnalyzer, Illumina, San Diego, CA). *De novo* assembly was conducted by Newbler (version 2.9; identity 98) (http://www.454.com). Of the assembled *B. calyciflorus* 140,587 contigs, 14 mitochondrial contigs were obtained. After a manual curation of 14 contigs with Consed (version 19.0) (http://www.phrap.org/consed/consed.html), two contigs were finally obtained to the mitochondrial DNAs of *B. calyciflorus*.

The complete mitochondrial genomes of *B. calyciflorus* the Netherlands strain were 27,698 bp (mitochondrial DNA I; GenBank no. MN457951) and 9,906 bp (mitochondrial DNA II; GenBank no. MN457952) in size. The direction of 13 protein-coding genes (PGCs) of *B. calyciflorus* was identical to those of *B. calyciflorus* China strain (Nie et al., 2016). Of 13 protein-coding genes (PCGs), three genes (*ND1*, *ND5*, and *ND3*) had incomplete stop codons. Furthermore, the start codon of *ND2*, *CO2*, and *CO3* and *ND4* genes was ATT, GTG, and ATA, respectively, while the start codon of other PCGs was ATG. The base composition of 13 PCGs in the Netherlands strain of *B. calyciflorus* mitogenome showed 31.1% for A, 37.6% for T, 16.5% for C, and 14.8% for G, respectively. The mitochondrial genome A + T base composition (72.0%) of 13 PCGs was higher than G + C (28.0%), whereas the complete mitochondrial genome A + T base composition (68.7%) was higher than G + C (31.3%).

The placement of *B. calyciflorus* in the genus *Brachionus* with 13 PGCs was shown in Figure 1. Two strains of *B. calyciflorus* were clustered closely to the freshwater rotifer *Brachionus rubens*. The gene order and contents of 13 PGCs of both *B. calyciflorus* were identical but tRNA-Ala and tRNA-Cys were rearranged in the mitochondrial DNA I, respectively. Particularly, tRNA-Leu and *16S rRNA* were additionally located in the mitochondrial DNA I in *B. calyciflorus* (the Netherlands strain), compared to China strain, while *B. calyciflorus* China strain had nine extra tRNAs (e.g. tRNA-Ala and tRNA-Arg for mitochondrial DNA I and tRNA-Met, tRNA-Tyr, tRNA-Val, tRNA-Glu, tRNA-Trp, tRNA-His, and tRNA-Pro for mitochondrial DNA II), compared to the Netherlands strain. This indicates that the rearrangement and additional copies of tRNAs is likely occurring in sporadic manner in the genus *B. calyciflorus* species complex.

**Figure 1.**
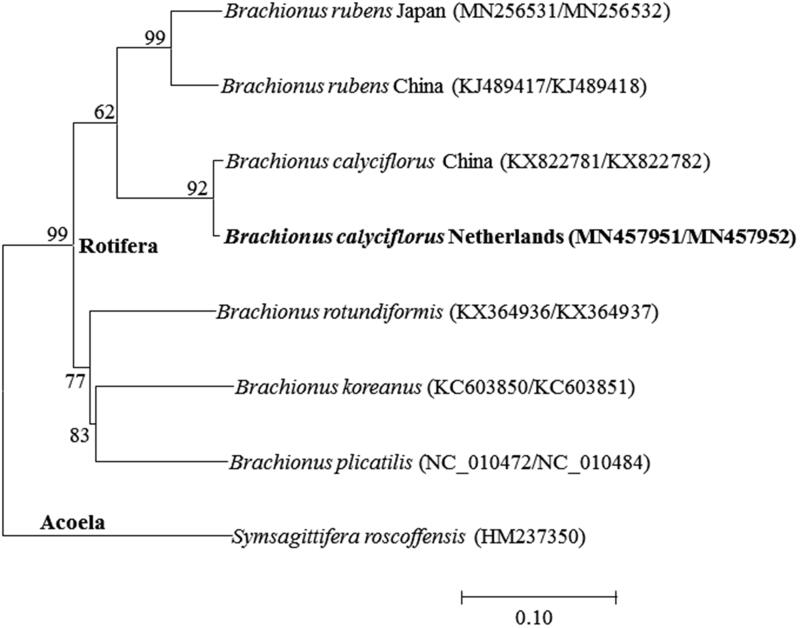
Phlyogenetic analysis of mitochondrial DNA. We conducted a comparison of the 13 mitochondiral DNA genes of Acoela and Rotifera. The 13 mitochondrial DNA genes were aligned by ClustalW. Maximum-likelihood analysis was performed by Mega software (ver. 10.0.1) with LG + G + I model. The rapid bootstrap analysis was conducted with 1000 replications with 48 threads running in parallel. The Acoela served as outgroup. -Ln = 24394.24.
